# Successful treatment of nonocclusive mesenteric ischemia in a reconstructed jejunum after esophagectomy and remnant gastric tube resection: a case report

**DOI:** 10.1186/s40792-023-01726-4

**Published:** 2023-08-10

**Authors:** Koichi Okamoto, Hiroto Saito, Mari Shimada, Takahisa Yamaguchi, Toshikatsu Tsuji, Hideki Moriyama, Jun Kinoshita, Keishi Nakamura, Itasu Ninomiya, Hiroyuki Takamura, Noriyuki Inaki

**Affiliations:** 1 Department of Gastrointestinal Surgery, Kanazawa University, 13-1 Takara-Machi, Kanazawa, Ishikawa 920-8641 Japan; 2 Department of General and Digestive Surgery, Kanazawa Medical University Hospital, 1-1 Daigaku, Uchinadamachi, Ishikawa, Kahoku 920-0293 Japan; 3 Department of Gastroenterological Surgery, Ishikawa Prefectural Central Hospital, 2-1 Kuratsukihigashi, Kanazawa, Ishikawa 920-8530 Japan; 4 Department of Surgery, Fukui Prefectural Hospital, 2-8-1 Yotsui, Fukui, 910-0846 Japan

**Keywords:** Nonocclusive mesenteric ischemia, Esophagectomy, Jejunal necrosis, Conservative treatment, Gastric conduit

## Abstract

**Background:**

Nonocclusive mesenteric ischemia (NOMI), an ischemic bowel disease without a disruption of the mesenteric blood flow or strangulation of the mesentery or intestine, may cause a lethal clinical course. We report a very rare case of jejunal necrosis caused by NOMI in the pedicled mesentery of the reconstructed jejunum after remnant gastric tube resection for heterochronous gastric tube cancer after esophagectomy.

**Case presentation:**

An 80-year-old man visited our department with chief complaints of fever and appetite loss after 4 months from gastric tube resection and digestive reconstruction with pedicled jejunum. Contrast-enhanced computed tomography (CT) revealed impaired blood flow without torsion of the mesentery, severe wall thickness, and micro-penetration in the reconstructed jejunum and combined pyothorax in the right thoracic cavity. Esophagogastroduodenoscopy demonstrated extensive mucosal necrosis confined to the jejunum, which was elevated in the thoracic cavity. The jejunal necrosis due to NOMI occurring in the reconstructed jejunum was suspected, and lifesaving small bowel resection with right thoracotomy was considered necessary. However, radical operation with right thoracotomy was considered to be excessively invasive and not valid due to the patient’s poor physical status, advanced age, and presence of left adrenal metastasis from the remnant gastric cancer. Therefore, we selected the conservative treatment with fasting, transnasal drainage, and administration of antibiotics due to the patient’s intention. CT-guided right thoracic drainage for the intrathoracic abscess was needed 10 days after starting treatment and the inflammatory response rapidly improved. Follow-up CT and esophagogastroduodenoscopy revealed the improvement in the ischemic changes in jejunal mucosa without perforation. Intake was initiated at 20 days after symptom onset, and the patient was discharged at 40 hospital days without any complications and sequelae.

**Conclusions:**

To the best of our knowledge, this is the first case of NOMI occurring in the reconstructed jejunum after remnant gastric tube resection that was successfully treated with a conservative treatment. For NOMI, it is important to make appropriate diagnosis based on imaging findings and perform proper assessment of the patient’s condition. Conservative treatments may be also useful depending on the patient’s condition.

## Background

Acute mesenteric ischemia (AMI) is a life-threatening vascular emergency caused by an interruption of the blood supply to the small intestine, with a high mortality rate ranging from 50 to 75% [1–3]. AMI is generally classified into the following three different forms of mesenteric ischemia: acute occlusive mesenteric ischemia, nonocclusive mesenteric ischemia (NOMI), and venous thrombosis of the mesenteric–portal axis. NOMI is caused by the arteriosclerosis of the mesenteric artery and intermittent spasm of blood vessels in the mesentery [1]. The diagnostic criteria of NOMI are as follows: (1) an ischemic bowel disease without disruption of the mesenteric blood flow; (2) without a strangulation of the mesentery or intestine; and (3) without an arterial or venous thrombosis at the mesenteric vessels [4–7]. Since NOMI generally develops in the small intestine existing in the abdominal cavity, it is difficult to follow its pathological condition and endoscopic findings throughout the clinical course.

To the best of our knowledge, no case of NOMI in the reconstructed jejunum existing in thoracic cavity after esophagectomy has been reported. We describe herein a particularly rare case of NOMI in the reconstructed jejunum after remnant gastric tube resection for heterochronous gastric tube cancer after esophagectomy, which was successfully treated with a conservative treatment.

## Case presentation

**Fig. 1:**
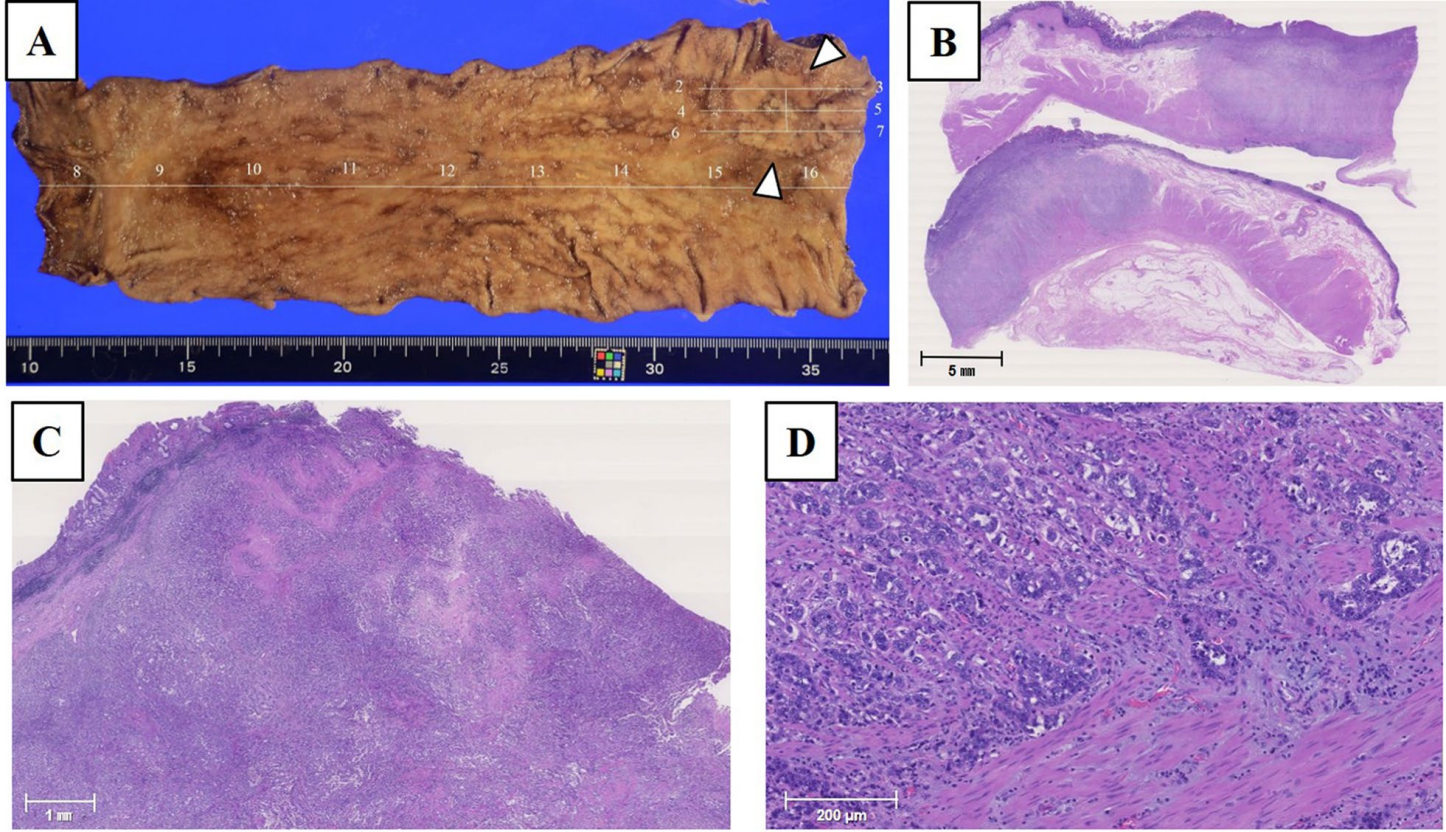
Pathological findings in a previously resected specimen of gastric tube cancer. **A** Macroscopic image of the resected gastric conduit with ulcerative gastric tube cancer with a diameter of 36 × 27 mm (white arrowhead). **B** Loupe view of gastric tube cancer in the resected gastric conduit. The invasion depth of gastric tube cancer was judged as pT4b-Trachea and it was pathologically suspected to have existed with residual cancer. **C** Microscopical findings showed the ulcerative tumor with bleeding and stromal fibrosis. **D** Moderate-to-poorly differentiated adenocarcinoma cells were observed and mainly composed of poorly differentiated components. Lymphatic and venous invasions are occasionally observed

**Fig. 2:**
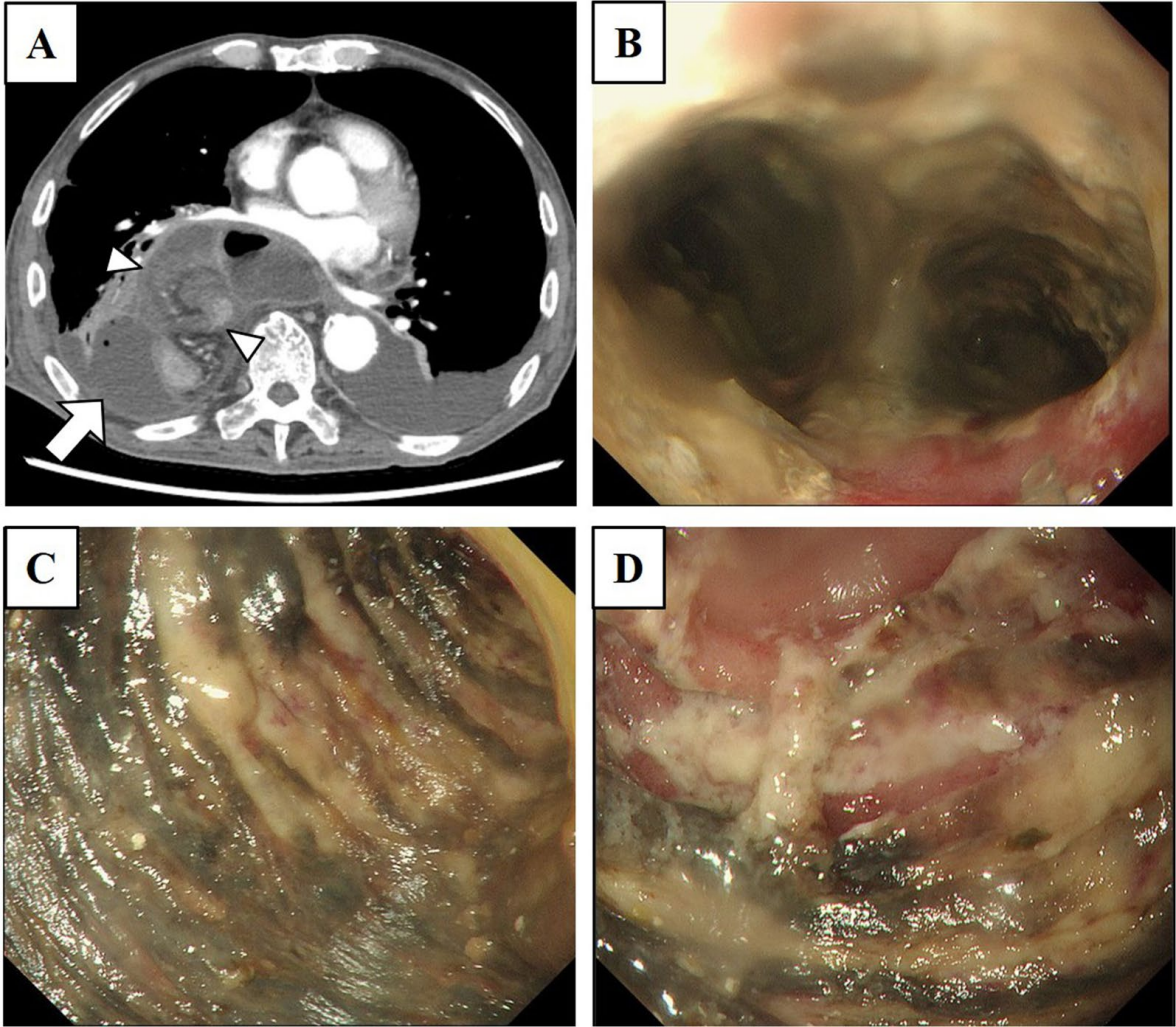
Imaging findings at the onset of nonocclusive mesenteric ischemia in the reconstructed jejunum. **A** Contrast-enhanced computed tomography showed diffuse wall thickening of the reconstructed jejunum elevated in the thoracic cavity with poor contrast enhancement (white arrowhead). The arterial and venous flow in mesentery of the reconstructed jejunum were maintained. A fluid correction with tiny free air was observed in the dorsal side of right thoracic cavity (white arrow). **B** In esophagogastroduodenoscopy, an apparent mucosal necrotic finding was observed at the most oral side of the reconstructed jejunum. **C** Segmental mucosal necrotic change was observed in the reconstructed jejunum elevated in thoracic cavity. **D** No necrotic finding was observed in the jejunum in abdominal cavity

**Fig. 3:**
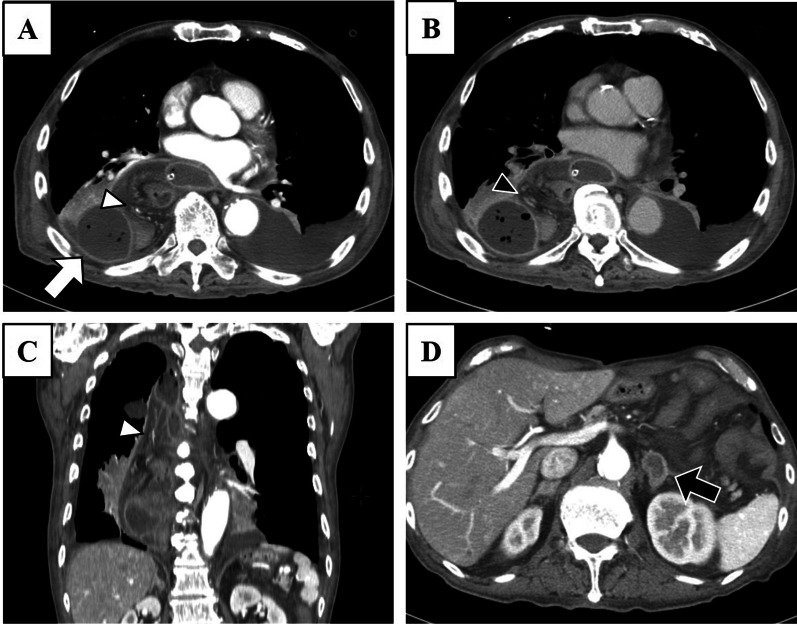
Contrast-enhanced computed tomography (CT) on the 9th hospital day. **A** Contrast-enhanced CT confirmed the arterial flow in the mesentery of the reconstructed jejunum (white arrowhead) and the intrathoracic abscess formation was organized (white arrow). **B** In the venous phase, sufficient venous flow was confirmed without disruption or thrombus in the mesenteric vessels of the reconstructed jejunum that was elevated in the thoracic cavity (black arrowhead). The arterial and venous flows in mesentery of the reconstructed jejunum were maintained. **C** Sagittal CT image showed sufficient arterial blood flow in the reconstructed jejunum that was elevated in the thoracic cavity (white arrowhead). **D** Metastatic left adrenal tumor derived from the remnant gastric cancer could be seen on the CT image

**Fig. 4:**
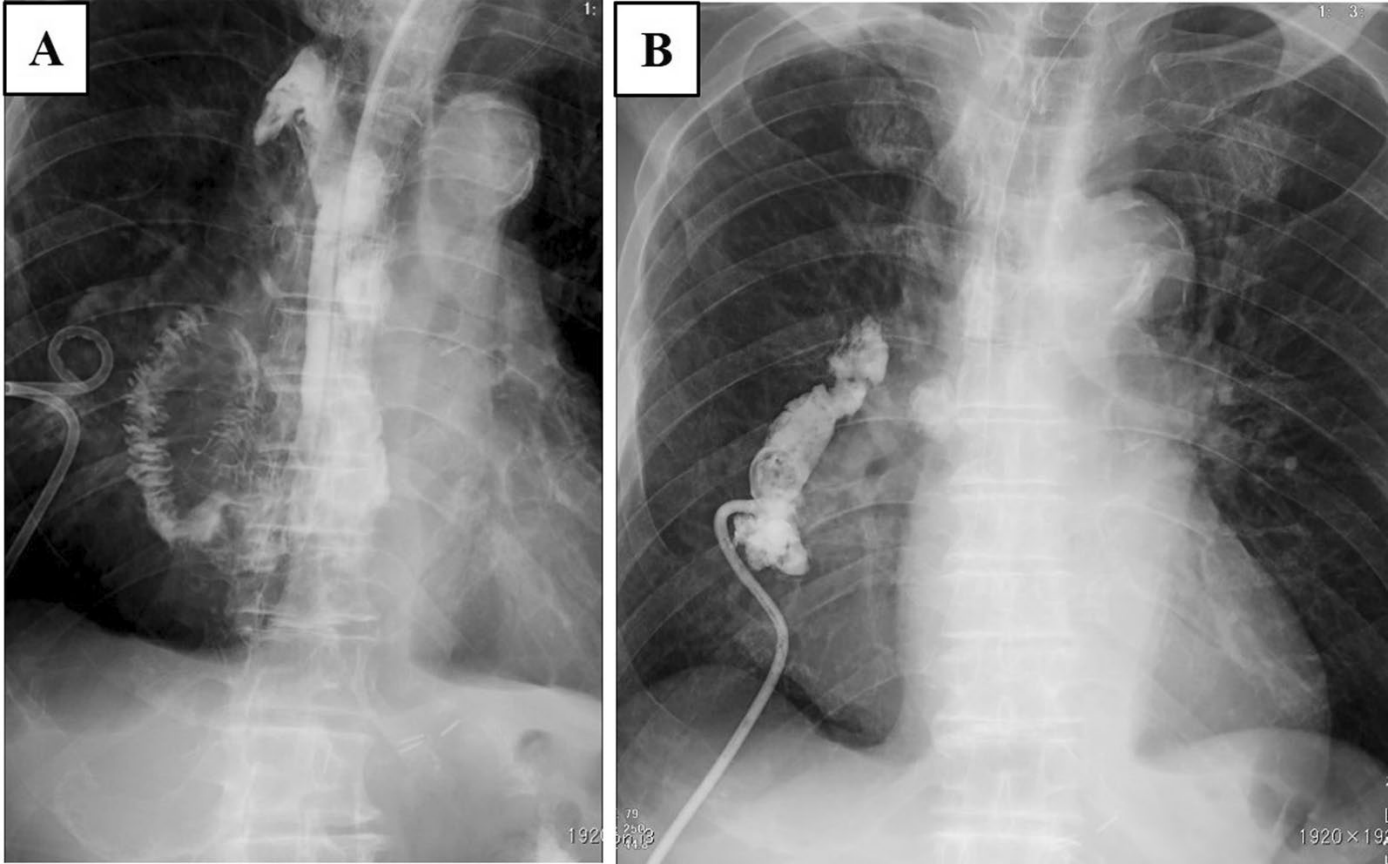
Radiographic image after the right thoracic drainage. **A** Esophagography revealed no evidence of perforation or interference in the passage at the reconstructed jejunum. **B** Fluoroscopy through the right thoracic drain showed no communication with the gastrointestinal lumen

**Fig. 5:**
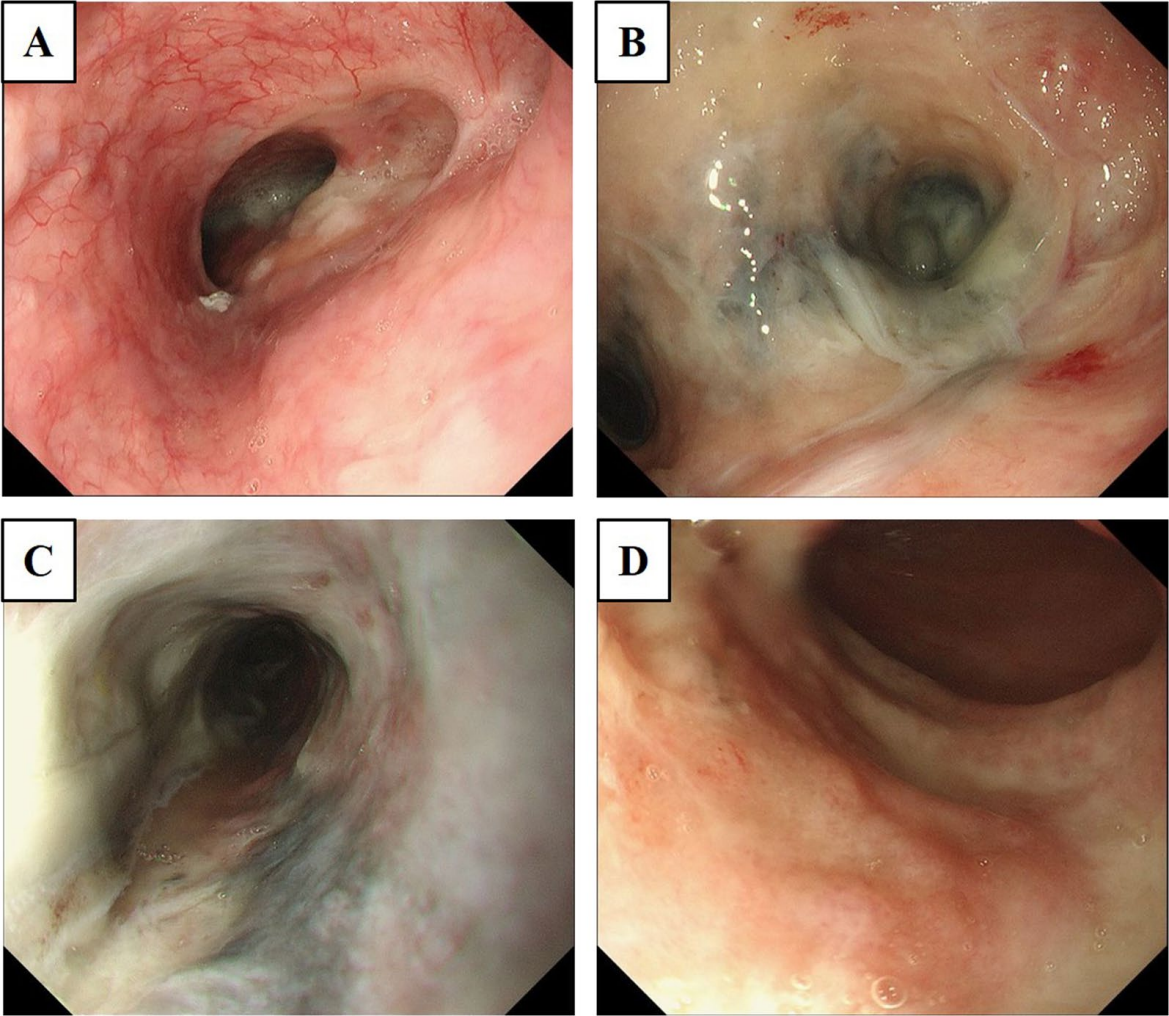
Findings of follow-up esophagogastroduodenoscopy at 20 days after treatment initiation. **A** Abnormal changes, including mucosal damage, and the stricture were not observed at the remnant esophagus and esophago-jejunal anastomosis. **B** Apparent improvement of the necrotic change at the most oral side of the reconstructed jejunum was observed. **C** Segmental mucosal necrotic change in the reconstructed jejunum that was elevated in the thoracic cavity improved. **D** Ischemic change at the esophageal hiatus also tended to improve and no ischemic changes were seen in the reconstructed jejunum in the abdominal cavity throughout the treatment course

An 80-year-old man who had previously undergone curative thoracoscopic esophagectomy for esophageal cancer 7 years earlier then underwent gastric conduit resection and digestive reconstruction by intrathoracic anastomosis using pedicled jejunum at the upper mediastinum for heterochronous gastric tube cancer (M-7-O, type 2, 36 × 27 mm, por-tub2, pT4b-Trachea, pN0M0H0P0CYX, pStage IIIa, PM0 DM0 RM1 Cur C) (Fig. 1). No direct invasion to other organs or apparent distant metastasis was observed in preoperative evaluation. However, the operation resulted in incomplete resection due to the residual cancer tissue on the tracheal wall. He had no postoperative complication, and he had received adjuvant chemotherapy using peroral S-1 (oral 5-fluorouracil prodrug). At 4 months after the surgery, he visited our hospital with complaints of ongoing fever, appetite loss, and nausea from 10 days ago. On examination, his vital signs were almost within the normal range, except for the mild fever. Contrast-enhanced computed tomography (CT) showed diffuse wall thickening of the reconstructed jejunum that was elevated in the thoracic cavity with poor contrast enhancement. In the arterial and venous phases, the mesenteric vessels of the reconstructed jejunum were clearly visualized, and no disruption or thrombus was observed. Fluid retention was observed in the reconstructed jejunum, but no torsion or strangulation of the intestinal tract or mesentery was evident. A small amount of fluid collection and free air was observed in the dorsal side of the right thoracic cavity (Fig. 2A). Esophagogastroduodenoscopy (EGD) revealed an apparent segmental mucosal necrotic finding in the reconstructed jejunum (Fig. 2B, C). Contrarily, no necrotic finding was observed in the jejunum in the abdominal cavity (Fig. 2D). He was suspected as having jejunal necrosis due to NOMI localized in the reconstructed jejunum with or without the penetration of the jejunum. It was suggested that small bowel resection with right thoracotomy is necessary as a lifesaving treatment. However, radical operation with right thoracotomy was considered to be excessively invasive due to the patient’s poor physical status, advanced age, and presence of left adrenal metastasis from the remnant gastric cancer that was detected on the CT image. In addition, the patient did not accept any invasive treatments. Therefore, we selected the conservative treatment with fasting, transnasal drainage, and antibiotic administration. Follow-up CT revealed the improvement of the ischemic and edematous changes in the reconstructed jejunal wall and a right intrathoracic abscess was suspected in the dorsal side of right thoracic cavity (Fig. 3A). The enhancement of the arteries and veins in the mesentery of the reconstructed jejunum was sufficiently maintained (Fig. 3B, C). A left adrenal metastasis from the remnant gastric cancer was again clearly detected (Fig. 3D). CT-guided right thoracic drainage for the intrathoracic abscess was performed 10 days after the start of treatment. After this additional procedure, his inflammatory response rapidly improved. Esophagography revealed no evidence of perforation or interference in the passage at the reconstructed jejunum (Fig. 4A). Fluoroscopy through the right thoracic drain showed no communication with the gastrointestinal lumen (Fig. 4B). Follow-up EGD revealed an apparent improvement of the necrotic findings in jejunal mucosa, which previously showed mucosal necrosis (Fig. 5). Oral intake was initiated at 20 days after disease onset and the patient was discharged at the 40 hospital days without any complications and sequelae. He decided not undergo further anti-cancer treatment after discharge. Unfortunately, at 5 months after discharge, he died from the recurrence of remnant gastric cancer.

## Discussion

AMI refers to the sudden onset of small intestinal hypoperfusion due to factors, such as mesenteric arterial embolism (50%), mesenteric arterial thrombosis (15–25%), or mesenteric venous thrombosis (5–15%) [2, 4]. AMI is a time-critical emergency resulting in irreversible hypoperfusion of the mesenteric organs within a few hours, leading to a high mortality rate. Our case was diagnosed as NOMI because of the absence of any evidence of mesenteric arterial or venous thrombosis. Several risk factors of AMI, including heart failure, atrial fibrillation, coronary heart disease, arterial hypertension, and peripheral vascular disease, have been reported [1, 2, 4]. Our patient actually had many risk factors, including advanced age, hypertension, emphysema, past history of deep venous thrombosis, and severe arteriosclerosis.

The superior mesenteric artery (SMA) is a single central vessel with a vulnerable terminal vascular zone [4]. The large diameter and narrow take-off angle of the SMA contribute to its anatomical susceptibility to decreased blood flow in the SMA. In our case, digestive reconstruction was performed using the pedunculated jejunum with the 2nd and 3rd jejunal arteries and veins branching from the SMA as feeding vessels. Therefore, the angle of the rising of the feeding vessels was toward the cranial side, which may be one of the causes of NOMI onset.

An acute complete circulatory disruption of the intestine leads to irreversible mucosal ischemia and gangrene of the intestinal wall due to prolonged hypoperfusion and increased intestinal pressure. The collapse of the mucosal barrier further contributes to bacterial translocation. In addition, bacterial infiltration leads to peritonitis, ileus, sepsis, and multiorgan failure, leading to a lethal clinical course [4, 8]. The patient's favorable goal is considered to be the improvement of the intrathoracic mesenteric blood flow by the sufficient intravenous fluid maintenance, with the aggressive supportive treatments of fasting, transnasal drainage for intestinal rest, and administration of broad-spectrum antibiotics to prevent bacterial translocation.

According to a national cancer registry in Japan, reconstructions using the jejunum accounted for 5.2% of all esophageal cancer resections [9]. Ninomiya et al. reported the usefulness of pedunculated jejunum reconstruction in the thoracic cavity as a gastrointestinal reconstruction method for patients who had undergone esophagectomy and total gastrectomy for esophageal cancer combined with gastric cancer or remnant stomach cases [10]. The same reconstruction method was performed after remnant gastrectomy in the present case without any postoperative complications.

Akiyama et al. reported a case of obstruction of the reconstructed bowel due to an incarcerated hiatal hernia after esophageal cancer surgery [11]. In our case, gastrointestinal passage disorder may occur at the esophageal hiatus, but physical stricture at the esophageal hiatus was absent in the imaging studies. However, the endoscopic findings of mucosal necrosis in the reconstructed jejunum were limited to the elevated jejunum in the thoracic cavity, and no ischemic findings were observed in the reconstructed jejunum in the abdominal cavity. The possibility that compression or bending of the mesentery at the esophageal hiatus may have caused NOMI cannot be ruled out.

Cases of a strangulated ileus in the elevated jejunum after total gastrectomy with Roux-en-Y reconstruction have been reported [12, 13]. In these cases, resection of the strangulated necrotic small intestine existing in the abdominal cavity and re-reconstruction of the gastrointestinal tract were required. In addition, Miura et al. reviewed the data of patients with NOMI occurring in a wide range of gastrointestinal tracts, including reconstructed gastric conduit that developed early after esophageal cancer surgery [14]. Among the reported cases, no cases of intestinal reconstruction in the thoracic cavity similar to our case were observed. The reconstructed jejunum can undergo ischemic change when it is twistedly elevated or pressed hard at the hiatus or when the mediastinal space is narrow for the conduit. In such cases, ischemic changes develop gradually and result in anastomotic stenosis or conduit stricture. However, in the case reported here, the onset of symptoms was rather sudden, and thus, the patient was diagnosed with NOMI.

Contrast-enhanced CT plays an essential role for the diagnosis and differential diagnoses of NOMI [4, 6, 7]. Moreover, interventional catheter angiography is recommended for stable patients with NOMI, as it enables diagnosis and treatment with the selective application of vasodilators into the SMA that can successfully interrupt the generalized vascular spasm. In addition, Miyazawa et al. reported that “time from CT to injecting vasodilator” was the only factor of survival for NOMI patients. Considering that NOMI is a pathological condition that does not necessarily lead to irreversible intestinal necrosis, some cases in the acute phase can be treated by conservative approaches by devising systemic management. In this case, given the patient’s stable vital signs, we thought that the pathologic condition improved by administration of intravenous fluid. In addition, as the presence of left adrenal gland metastasis was confirmed at the time of onset, the patient had no desire to receive invasive and aggressive treatment, including interventional catheter angiography, and continuous injection of vasodilators, such as papaverine and PGE1. Contrarily, the risk of in-hospital mortality is high in severe patients with a sequential organ failure assessment (SOFA) score of ≥ 8 at the time of NOMI onset [3]. In our case, there were no major disturbances in vital signs at disease onset, the SOFA score on blood tests was as low at two points, and the administration of circulatory agonists was unnecessary.

Due to surgical complexity and our patient’s poor general condition and wishes, surgery was not selected. In this situation, radical surgery via thoracotomy should ideally be performed, in which the reconstructed jejunum is resected and digestive re-reconstruction is performed using the ileocolon or left colon with or without additional vascular anastomosis depending on the situation [15]. The surgical procedure itself can be highly invasive, and it is not necessarily easy to re-reconstruct the gastrointestinal tract after resection of the necrotic reconstructed jejunum. Conservative treatment is considered to have a great advantage, because the surgery may not ultimately be lifesaving. Conversely, we considered that active drainage of the contents in the reconstructed jejunum can improve organ blood supply from the decompression in the reconstructed jejunum and subsequently regenerate the gastrointestinal wall if the blood supply of the reconstructed jejunum is maintained and complete necrosis has not occurred. In this case, active gastrointestinal drainage using a nasogastric tube was effective. In addition, a clinical trial on aspirin administration for suppressing the onset of cardiovascular events showed that low-dose aspirin administration increased the risk of gastrointestinal bleeding but had a poor effect on suppressing cardiovascular event [16]. We judged that the usefulness of antiplatelet agents in suppressing the occurrence of NOMI was poor, and therefore, we did not administer low-dose aspirin or anticoagulants in this case, although arteriosclerosis was very serious.

Since NOMI generally develops in the small intestine existing in the abdominal cavity, a large part of the intestines is generally inaccessible via endoscopy [17]. In this point of view, our case was an extremely rare that NOMI locally occurred at the reconstructed jejunum and the entire healing process could be observed. Although an extremely rare and critical disease developed, our patient survived because of an accurate diagnosis and appropriate management.

## Conclusions

We report the natural course of NOMI and a rare case of NOMI occurring in the reconstructed jejunum that was elevated in the thoracic cavity, in which the conservative approach and observation with EGD could be fortunately possible.

## Data Availability

All data generated during this report are included in this published article.

## Abbreviations

AMI: Acute mesenteric ischemia
NOMI: Nonocclusive mesenteric ischemia
CT: Computed tomography
EGD: Esophagogastroduodenoscopy
SMA: Superior mesenteric artery
SOFA: Sequential organ failure assessment
